# The obese population’s views on the symptoms and risks of chronic venous insufficiency - 2 (OBVIOUS-2) cross-sectional survey

**DOI:** 10.1177/02683555241284179

**Published:** 2024-09-17

**Authors:** Matthew A Popplewell, Sindoora Mahesh, Sandip Nandra, Maciej Juszczak, Helen Ashby, Michael L Wall

**Affiliations:** 1 Black Country Vascular Network, 7714 Dudley Group of Hospitals NHS Trust, Dudley, UK; 2 Institute of Applied Health Research, 1724 University of Birmingham, Birmingham, UK; 3 Academic Vascular Surgery, 5994 Newcastle University, Newcastle upon Tyne, UK; 4 Department of Vascular Surgery, 1732 University Hospitals Birmingham NHS Foundation Trust, Birmingham, UK; 5 Weight Management Services, 7714 Dudley Group of Hospitals NHS Trust, Dudley, UK

**Keywords:** Chronic venous disease, obesity

## Abstract

**Introduction:**

Individuals with high body mass index (BMI) are more likely to have symptomatic LLVD than age matched populations with normal BMI. National priorities in venous disease set by the James Lind Alliance focus on improving access to healthcare and patient education. The aims of this study are to determine patient knowledge and potential burden of LLVD in a population of patients attending a UK, regional weight management service.

**Methods:**

A postal questionnaire containing 12 questions relating to LLVD and obesity was distributed to the active list of patients under the weight management medical service at Dudley Group of Hospitals between May 2022-23. Respondents were provided with a stamped, addressed envelope to return the questionnaire. Ethical approval was granted by the Hampshire Research & Ethics Committee.

**Results:**

Some 367 questionnaires were distributed to patients currently enrolled in specialist weight management services. 103 complete responses were received (28%), Most patients were between 50 and 70 years of age. 25% of patients already had a formal diagnosis of LLVD, with a further 84 (82%) reported signs or symptoms which may be related to LLVD. Almost half (49/103, 48%) had concerns over their skin quality with a similar proportion (25/103, 51%) having sought medical help. The majority (71/103, 69%) were unaware of the association between obesity and LLVD. Twelve participants had education regarding simple adjuncts designed to improve symptoms and/or prevent ulceration (emollients, dressings, stockings, or leg elevation). Four participants had previously undergone treatment for varicose veins.

**Conclusion:**

In a population of patients accessing weight management services, we have demonstrated that a quarter of patients have already received a diagnosis of LLVD, however there is for a greater undiagnosed burden of LLVD in part due to lack of patient and possibly clinician awareness.

## Introduction

### Background

Lower limb venous disease (LLVD) is known to affect up to 40% of the general population. Leg ulceration is the most severe manifestation of LLVD, and the care of leg wounds is estimated to cost the National Health Service (NHS) in the UK around £2 billion per year.^1^ There is a known association between LLVD and obesity (defined as a body mass index greater than 30 kg/m^2^ in the UK) in that patients who are obese are more than twice as likely to develop venous reflux (odds ratio [OR] 2.1, 95% CI 1.0–4.4).^2^ Obesity has been declared a public health crisis by the World Health Organisation (WHO) who have recently predicted that by 2030, one billion adults globally will be obese. This has stimulated the publication of the ‘WHO acceleration plan to stop obesity’ this year.^3^ In the UK one in four adults are currently living with obesity.^4^ Based on these numbers, the incidence of concurrent obesity and LLVD is likely to rise and manifest as an increased workload for vascular services and significant healthcare costs worldwide.

The interplay between LLVD and obesity is likely to be complex due to mechanical (increased intra-abdominal pressure, increased femoral vein pressure and diameter, decreased flow) and biochemical (inflammatory) interactions. The effect of OBesity on Venous Impedance and Outflow measured by UltraSound (OBVIOUS) study reported that increased abdominal fat reduced venous return in the femoral vein.^5^ Many epidemiological studies have demonstrated the link between obesity and the presence of varicose veins and chronic venous insufficiency.^6^

In 2021, The James Lind Alliance (JLA) Venous Top 10 priorities was published,^7^ the top two being listed as;

• How can all patients be given the opportunity to access the specialist assessment and treatment they need?
• How can awareness and education of venous disease be improved?

Based on the increasing levels of obesity, the potential for increasing volumes of significant LLVD and the JLA priorities we aim to study the prevalence, impact, and knowledge of LLVD using a novel questionnaire in a population of obese patients who are currently accessing specialist weight management services within the Black Country, UK.

This report was compiled using the Consensus-based checklist for the Reporting Of Survey Studies (CROSS).^8^

## Methods

Prior to dissemination a specific, cross-sectional questionnaire was developed by a vascular surgeon (MW), a clinical pathologist (HA) and a research fellow (SJ). The questionnaire was designed to capture relevant information pertaining to the prevalence, severity, and specific characteristics of potentially undiagnosed LLVD. The questionnaire also captured patient perspectives or concerns regarding their skin health, pre-existing diagnoses, and education regarding potential LLVD. This consisted of 12 questions and used a combination of dichotomous questions, multiple choice elements and a Likert scale. A full copy of the questionnaire is available in the supplementary materials. The questionnaire was assessed by the group, as well as members of the Black Country Research & Development (R&D) team for suitability and readability. A pilot of the questionnaire was trialled by members of the R&D team as pilot to screen for errors and improve readability.

A prospective, cross-sectional survey was performed of current patients enrolled in The Dudley Group of Hospitals (DGOH) weight management services between May 2022 and May 2023. The target population was all patients over the age of 18 years who accessed specialist weight management services during the timeframe.

The Dudley Group of Hospitals (DGOH) weight management services accepts referrals from the Black Country Integrated Care Board (ICB) in the UK. Patients are referred based on the current National Institute for Health & Care Excellence (NICE) criteria for referral to specialist weight management for adults.^9^ The Black Country ICB covers a population of 1.26 million people around 20% are based in the DGOH catchment.^10^ The DGOH weight management services receive around 300 referrals per annum and has approximately 1100 patients currently active in the system.

Simple random sampling was performed by sending the questionnaire to participants by mail with a stamped, addressed envelope for return to the R&D department at DGOH. Data was then collated and entered into a Microsoft Excel© spreadsheet for analysis. Participants were anonymous, in that the returned questionnaire contained no patient identifiable information. Only one letter/questionnaire was sent to each household to prevent multiple participation.

A patient information sheet was included with the questionnaire explaining the aims and purpose of the research (see supplementary materials).

The study received ethical approval from the Hampshire Research & Ethics Committee on the 4^th^ April 2022 (22/SC/0075). The principles of Good Clinical Practice were closely adhered to.^11^ The favourable ethical approval letter is attached in the supplementary materials.

Survey data was analysed by a single author (SJ) using a password encrypted database held securely at the host R&D department (DGOH, UK). Incomplete questionnaires were included in the analysis, missing data is clearly stated in the results. Imputation was not used to account for missing data. Dichotomous data were expressed as a percentage and multiple‐choice elements were expressed as parts of the whole (%). Graphical illustrations were prepared using the Microsoft 365 package.

## Results

**Figure 1.**
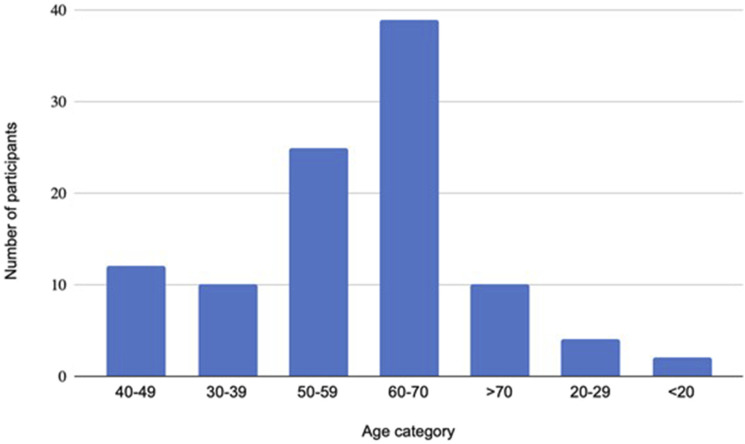
Age of respondents in The OBese population’s VIews on the symptoms and risks of chronic venOUS insufficiency - 2 (OBVIOUS-2) Cross-sectional Survey.

A total of 367 questionnaires were distributed to patients who enrolled in specialist weight management services in the survey period and a total of 103 (28%) questionnaire responses were received. The age of the respondents was widely spread; however, most were between 50 and 70 years old (Figure 1).

Participants were asked if skin quality on the lower leg was of concern (responses yes/no/unsure). Almost half of the respondents said yes (49/103, 47.6%) and a further proportion reported being ‘unsure’ (7/103, 6.8%). One person declined to answer.

When asked, 71/103 (68.9%) respondents were not aware or not sure of the association between obesity and skin changes in the context of LLVD.

**Figure 2.**
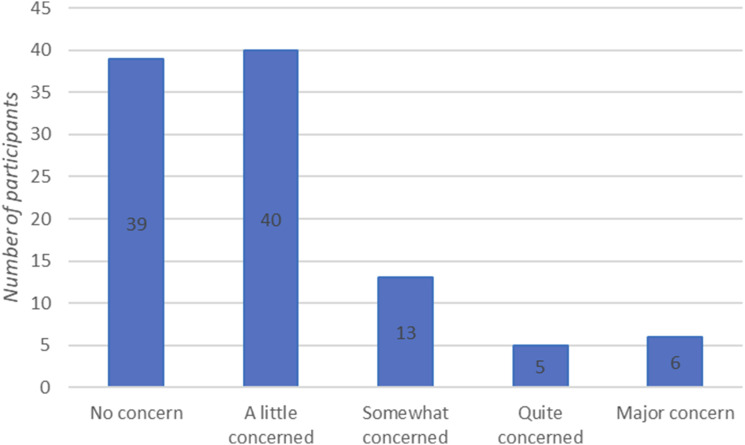
Participant ratings regarding their level of concern over the skin quality of their legs in The OBese population’s VIews on the symptoms and risks of chronic venOUS insufficiency - 2 (OBVIOUS-2) Crossectional Survey.

Participants were asked to rate their level of concern over the skin quality on their legs based on a Likert scale (ranging from no concern to major concerns. 64 participants (62%) reported having concerns regarding the quality of their skin (Figure 2).

If participants had concerns regarding their skin quality, they were then asked to elaborate on what these might be. 63 patients (61%) reported concerns, and these were often multiple in a single person. Factors were identified relating to appearance (46/103, 44.7%), pain/discomfort (42/103, 40.1%), sleep quality (28/103, 27.2%) and effects on mood/mental health (23/103, 22.3%) as well as daily life (28/103, 27.2%).

Of those that had concerns regarding their skin health less than half had sought medical attention (24/63, 38%). Participants were asked what sources of help they had received in managing their skin. 95 people responded. The majority had not sought any help for their skin or felt that it was not required (54/95, 57%). Thirty (30/95, 32%) reported visiting their primary care doctor, 9 (9%) had accessed online resources, 8 (8%) had received an information leaflet and 4 (4%) had sought alternative therapies (participants could select multiple responses).

Participants were then specifically asked if they had received advice if it related to common therapies for LLVD (emollients, dressings, elevation, and stockings). 59 responded and the majority had reported that no advice was given (27/59, 46%). Those that had reported being told about stockings (18/59, 31%), emollient (17/59, 29%), leg elevation (16/59, 27%) and dressings (9/59, 15%). Some reported being advised to take antibiotics (2/59, 3%) and diuretics (1/59, 2%) (participants could select multiple responses).

From the total cohort 84 respondents (81.6%) reported skin changes and/or symptoms which could be suggestive of LLVD. Several participants had multiple symptoms and signs with 59 (57%) having at least two features that could be suggestive of LLVD.

A quarter of respondents (26 of 102, 25%, one participant did not respond) reported having a previous specific diagnosis of LLVD (which included deep venous thrombosis (5/26, 19%), varicose veins (11/26, 42%) venous ulceration (2/26, 8%) or lymphoedema (14/26, 54%). A small proportion of patients were unsure if they had a previous diagnosis of LLVD (8/102, 8%).

When specifically asked about current symptoms pertaining to LLVD all but two people responded (Figure 4). The majority reported symptoms that could be secondary to LLVD (5 had an active ulcer, six reported having healed an ulcer, 35 had skin discolouration, 54 reported swelling, 12 had weeping, 57 reported muscle aches or cramping and 35 had skin burning, itching or pain). Only four participants reported having had a previous treatment for LLVD, all of which were for varicose veins (2/4 endothermal ablation and 2/4 open surgery).

## Discussion

The OBVIOUS-2 questionnaire has demonstrated that in a population of patients who access weight management services that there are a high proportion that currently have or could have LLVD. Around a quarter of patients had already received a formal previous diagnosis of LLVD. This is in keeping with findings of the Edinburgh Vein Study who reported a 13-years incidence of 23.6% in the obese population (compared to 6.1% in patients with normal weight).^2^ The survey response was 28% of the current enrolled weight management services population, this response rate would be widely considered as acceptable. With respect to this it is possible that the current burden of LLVD is over or underrepresented in this study. However, most patients (81.6%) reported signs and/or symptoms which may correlate to a diagnosis of LLVD. This may suggest that either the condition is underdiagnosed or there is some selection bias in our sample, in that people with potential leg problems may have been more likely to respond to our questionnaire.

It is concerning that over two-thirds of the patients were not aware of the association of LLVD and obesity, given that the incidence is around a quarter in such patients. The Edinburgh vein study reported longer term outcomes in 2013, and a decade later, awareness of this association was poor in our cohort.

Despite only a quarter of the surveyed population having had a formal diagnosis of LLVD, a larger proportion (62%) had concerns about the appearances of their skin, albiet the majority minor concerns. This may suggest that there is a proportion of patients with undiagnosed LLVD in our cohort. It is difficult to speculate if such patients have symptoms or if they do, that is sufficiently affecting their quality-of-life to warrant further investigation or treatment. We did not use formal, validated questionnaire such as the Aberdeen Varicose Vein Questionnaire (AVVQ)^12^ or the ChronIc Venous Insufficiency quality of life Questionnaire (CIVIQ-20),^13^ however, 27.2% of respondents reported sleep disturbance and 22.3% reported detriment to mood/mental health. If such skin changes were secondary to LLVD then these patients may qualify for treatment according to current national guidance in the UK.^14^

A large cohort of the surveyed population did not access healthcare. We did not specifically ask participants why they did not access healthcare. We postulate that this could be due to multiple factors such as difficulty accessing primary and secondary care, lack of patient and medical community education regarding the association between LLVD and obesity and other healthcare issues which may take precedent in this population. When patients did access healthcare and were given leaflets, online resources, and clinical advice they were still unaware of this association. A large patient and public health effort is therefore required to raise awareness of this association and that several treatments are available that could improve clinical outcomes and health related quality of life.^15^

Four participants reported that they had undergone previous surgical treatment for LLVD. We did not collate when such intervention was performed (it could have been performed at a lower patient weight). Currently there is debate regarding treatment efficacy and safety in obese patients with LLVD. A large review of over 60,000 patients undergoing venous procedures (endothermal ablation +/− foam/phlebectomies or endothermal ablation alone) reported a negative correlation with increasing BMI (>35) and CIVIQ-20.^16^ Interestingly the outcomes of patients with a BMI >45 were so poor that the group recommended weight loss prior to considering intervention. A much smaller, more recent study reported that those with a BMI >30 undergoing truncal ablation had higher rates of infection (3.0% vs 0.8% in lower BMI group) and had more residual pain (as measured by visual analogue scale) at 6 weeks following treatment^17^ but with good technical outcomes at 1-year (occlusion rates of 97.6%-100%) and no increased risk of bleeding or venous thromboembolism. The study did not report HRQoL or differentiate BMI >30 so it is difficult to comment on the proportion of patients with morbid obesity like those in this cohort.

### Limitations

Our survey may be prone to bias in that the sampled population likely represent the upper limit of patients with obesity. People with a BMI over 35 but less than 45 are not included in this survey. Therefore, the prevalence of LLVD and potential symptoms pertaining to LLVD may be over-estimated when considering the UK entire obese population.

Our survey response rate was 28%, which is congruent with experimental studies assessing response rates in population-based surveys. This does mean however, that a large proportion of our population remains unsampled. As discussed previously it would have been preferrable to use a validated, generic or disease specific quality of life assessment tools, but they were not included in the questionnaire.

Our survey was only conducted in one part of the UK where the DGOH serves around 450,000 patients. The prevalence of obesity is likely to vary in different parts of our country and in fact the world. It is impossible to know if the findings of this study are generalisable, but there are likely to be similarities.

## Conclusion

In a population of patients accessing weight management services, we have demonstrated that a quarter of patients have already received a diagnosis of LLVD, however there is likely to be a much higher undiagnosed burden of LLVD due to lack of awareness. Further work is required to highlight which patients could benefit from further assessment and/or treatment, and in what order.

## Supplemental Material

Supplemental Material - The obese population’s views on the symptoms and risks of chronic venous insufficiency - 2 (OBVIOUS-2) cross-sectional survey Supplemental Material for The obese population’s views on the symptoms and risks of chronic venous insufficiency - 2 (OBVIOUS-2) cross-sectional survey by Matthew A Popplewell, Sindoora Mahesh, Sandip Nandra, Maciej Juszczak, Helen Ashby and Michael L Wall in Phlebology

Supplemental Material - The obese population’s views on the symptoms and risks of chronic venous insufficiency - 2 (OBVIOUS-2) cross-sectional survey Supplemental Material for The obese population’s views on the symptoms and risks of chronic venous insufficiency - 2 (OBVIOUS-2) cross-sectional survey by Matthew A Popplewell, Sindoora Mahesh, Sandip Nandra, Maciej Juszczak, Helen Ashby and Michael L Wall in Phlebology
