# Supplementary material for: The obese population’s views on the symptoms and risks of chronic venous insufficiency - 2 (OBVIOUS-2) cross-sectional survey
Source: Phlebology. 2024 Sep 17;40(3):161–6. doi: 10.1177/02683555241284179 (PMC11951361; doi:10.1177/02683555241284179)
Supplement: Supplemental Material - The obese population’s views on the symptoms and risks of chronic venous insufficiency - 2 (OBVIOUS-2) cross-sectional survey [file sj-pdf-1-phl-10.1177_02683555241284179.pdf]

Supplementary Material 1 - OBVIOUS 2 patient questionnaire

Russells Hall Hospital
Dudley
West Midlands
DY1 2HQ

OBVIOUS 2 Questionnaire

Dear Volunteer,

We would like to invite you to participate in a piece of research being out by questionnaire here at Russells Hall Hospital which we hope might lead to an improvement in long term patient care.

The questionnaire has been designed by Mr Michael Wall and **Dr Helen Ashby** to look at the potential problems around lower leg symptoms, skin colour changes, skin ulceration, and their links to being overweight.

There is strong evidence that patients who have a higher body weight can have skin colour changes, unpleasant symptoms or even ulcers in their legs. However, there is very little known about the rate at which these skin problems occur in the population and patient's perception of how these skin problems can impact their lifestyle and day to day activities.

The purpose of this study is to assess, firstly, whether you have experienced any skin problems on your legs. Secondly your awareness of the issue and its impact. Thirdly, investigate your ideas, beliefs, or worries regarding your own experience with your skin.

To take part, we ask that you fill out the following questions and return the form using the pre-paid envelope enclosed. All information collected in this survey will be used only for research purposes and will be kept confidential. There will be no connection or reference made to you in all future publications of this study and we request that you **DO NOT** write your name or any other identifiable information on this form to ensure it remains anonymous.

☐ Please tick the box to show that you have read the above information, understand, and are happy for the information you have provided in the following survey to be used for research purposes.

This questionnaire is completely anonymous and the answers to these questions will in no way affect the care provided to you.

1. Please select your age range:

❏ <20
❏ 20-29
❏ 30-39
❏ 40-49
❏ 50-59
❏ 60-70
❏ >70

2. Have you ever had concerns over the skin quality of your legs?

❏ Yes
❏ No
❏ Not sure

3. Were you aware of an association between obesity and skin changes?

❏ Yes
❏ No
❏ Not sure

4. Mark on the scale below how concerned you are about the skin quality on your legs?

❏ 1 - No concern
❏ 2 - A little concerned
❏ 3 - Somewhat concerned
❏ 4 - Quite concerned
❏ 5 - Major concern

5. If you have concerns regarding your skin health what are they? Please select all that apply

❏ Appearance
❏ Pain/discomfort
❏ Effect on your daily life
❏ Effect on mood and mental health
❏ Sleep quality

- ❏ None
- ❏ Other: ____ (please specify)

6. Have you ever sought help regarding the skin quality on your legs?

- ❏ Yes
- ❏ No

7. What sources of help have you had in managing your skin? Please select all that apply

- ❏ Medical advice (e.g. from your GP/doctor)
- ❏ Information leaflets
- ❏ Online resources
- ❏ Alternative therapies
- ❏ None
- ❏ None required
- ❏ Other: ____ (please specify)

8. If you answered yes to question 7 - Were you ever educated about the use of the following treatments? Please select all that apply

- ❏ Emollients
- ❏ Dressings
- ❏ Elevation
- ❏ Stockings
- ❏ None
- ❏ Other: ____ (please specify)

9. Have you ever had a diagnosis of venous disease e.g. Deep venous thrombosis (DVT), varicose veins, venous ulcer...

- ❏ Yes
- ❏ No

- ❑ Not sure

10. If you answered yes to Question 9 – Which skin or venous conditions, have you been diagnosed with? Please select all that apply

- ❑ Varicose veins (dilated/ enlarged blue veins commonly in legs)
- ❑ Lymphedema (Fluid build-up in legs)
- ❑ Peripheral vascular disease (narrowing in blood vessels)
- ❑ Deep vein thrombosis (Blood clot in your leg vein)
- ❑ Not sure
- ❑ Other: ______________________________________ (please specify)

11. Do you have any of these symptoms affecting your legs? Please select all that apply

- ❑ An active ulcer (break in the skin that takes > 6 weeks to heal)
- ❑ Healed ulcer (break in the skin that takes > 6 weeks to heal)
- ❑ Skin discolouration (blue/brown/black colour changes of legs)
- ❑ Swelling (fluid build-up in legs)
- ❑ Weeping (fluid leaking from your skin)
- ❑ Skin itching/burning/pain
- ❑ Muscle aches/cramping
- ❑ None
- ❑ Other: ______________________________________ (please specify)

12. Have you ever had surgery for varicose veins? Please tick all that apply

- ❑ No
- ❑ Yes – Laser/ Heating/Burning Therapy
- ❑ Yes – Foam
- ❑ Yes – Stripping

Thank you for completing this questionnaire

Please return in the stamped addressed envelope

Research and Development Contact Details:

Principal Investigator: Mr Michael Wall (Vascular and Venous Surgeon)
Co- Investigator: Dr Helen Ashby (Consultant Clinical Pathologist)

Tel: 01384 456111 Ext: 1024

Email: dgft.research.rhh@nhs.net

Address:
Research & Development Department
1st Floor, North Wing,
Russells Hall Hospital
Pensnett Road,
Dudley
DY1 2HQ
