# Supplementary material for: The obese population’s views on the symptoms and risks of chronic venous insufficiency - 2 (OBVIOUS-2) cross-sectional survey
Source: Phlebology. 2024 Sep 17;40(3):161–6. doi: 10.1177/02683555241284179 (PMC11951361; doi:10.1177/02683555241284179)
Supplement: Supplemental Material - The obese population’s views on the symptoms and risks of chronic venous insufficiency - 2 (OBVIOUS-2) cross-sectional survey [file sj-pdf-2-phl-10.1177_02683555241284179.pdf]

South Central - Hampshire A Research Ethics Committee
Temple Quay House
2 The Square
Temple Quay
Bristol
BS1 6PN
Telephone: 0207 104 8196

04 April 2022

Mr Michael Wall
Consultant Vascular Surgeon
The Dudley Group NHS Foundation Trust
Black Country Vascular Network
Russell Hall Hospital
Dudley
DY1 2HQ

Dear Mr Wall

**Study title:**	**The OBese Populations VIews on the Risk of Chronic VenOUS Insufficency within the population. OBVIOUS 2 study**
**REC reference:**	**22/SC/0075**
**IRAS project ID:**	**300134**

Thank you for responding to the Proportionate Review Sub-Committee's request for changes to the documentation for the above study.

The revised documentation has been reviewed and approved on behalf of the PR sub-committee.

Confirmation of ethical opinion

On behalf of the Research Ethics Committee (REC), I am pleased to confirm a favourable ethical opinion for the above research on the basis described in the application form, protocol and supporting documentation as revised.

Good practice principles and responsibilities

The UK Policy Framework for Health and Social Care Research sets out principles of good

practice in the management and conduct of health and social care research. It also outlines the responsibilities of individuals and organisations, including those related to the four elements of research transparency:

1. registering research studies
2. reporting results
3. informing participants
4. sharing study data and tissue

Conditions of the favourable opinion

The REC favourable opinion is subject to the following conditions being met prior to the start of the study.

Confirmation of Capacity and Capability (in England, Northern Ireland and Wales) or NHS management permission (in Scotland) should be sought from all NHS organisations involved in the study in accordance with NHS research governance arrangements. Each NHS organisation must confirm through the signing of agreements and/or other documents that it has given permission for the research to proceed (except where explicitly specified otherwise).

Guidance on applying for HRA and HCRW Approval (England and Wales)/ NHS permission for research is available in the Integrated Research Application System.

For non-NHS sites, site management permission should be obtained in accordance with the procedures of the relevant host organisation.

Sponsors are not required to notify the Committee of management permissions from host organisations.

Registration of Clinical Trials

All research should be registered in a publicly accessible database and we expect all researchers, research sponsors and others to meet this fundamental best practice standard.

It is a condition of the REC favourable opinion that **all clinical trials are registered** on a publicly accessible database within six weeks of recruiting the first research participant. For this purpose, ‘clinical trials’ are defined as:

- clinical trial of an investigational medicinal product
- clinical investigation or other study of a medical device
- combined trial of an investigational medicinal product and an investigational medical device
- other clinical trial to study a novel intervention or randomised clinical trial to compare interventions in clinical practice.

Failure to register a clinical trial is a breach of these approval conditions, unless a deferral has been agreed by the HRA (for more information on registration and requesting a deferral see: Research registration and research project identifiers).

If you have not already included registration details in your IRAS application form you should notify the REC of the registration details as soon as possible.

Publication of Your Research Summary

We will publish your research summary for the above study on the research summaries section of our website, together with your contact details, no earlier than three months from the date of this favourable opinion letter.

Should you wish to provide a substitute contact point, make a request to defer, or require further information, please visit: https://www.hra.nhs.uk/planning-and-improving-research/application-summaries/research-summaries/

N.B. If your study is related to COVID-19 we will aim to publish your research summary within 3 days rather than three months.

During this public health emergency, it is vital that everyone can promptly identify all relevant research related to COVID-19 that is taking place globally. If you haven't already done so, please register your study on a public registry as soon as possible and provide the REC with the registration detail, which will be posted alongside other information relating to your project. We are also asking sponsors not to request deferral of publication of research summary for any projects relating to COVID-19. In addition, to facilitate finding and extracting studies related to COVID-19 from public databases, please enter the WHO official acronym for the coronavirus disease (COVID-19) in the full title of your study. Approved COVID-19 studies can be found at: https://www.hra.nhs.uk/covid-19-research/approved-covid-19-research/

It is the responsibility of the sponsor to ensure that all the conditions are complied with before the start of the study or its initiation at a particular site (as applicable).

After ethical review: Reporting requirements

The attached document "After ethical review – guidance for researchers" gives detailed guidance on reporting requirements for studies with a favourable opinion, including:

- Notifying substantial amendments
- Adding new sites and investigators
- Notification of serious breaches of the protocol
- Progress and safety reports
- Notifying the end of the study, including early termination of the study
- Final report
- Reporting results

The latest guidance on these topics can be found at https://www.hra.nhs.uk/approvals-amendments/managing-your-approval/.

Ethical review of research sites

The favourable opinion applies to all NHS/HSC sites taking part in the study, subject to management permission being obtained from the NHS/HSC R&D office prior to the start of the study (see "Conditions of the favourable opinion" above).

Approved documents

The documents reviewed and approved by the Committee are:

Document	*Version*	*Date*
IRAS Application Form [IRAS_Form_16022022]		16 February 2022
Letter from sponsor [Sponsorship letter]		22 March 2022
Non-validated questionnaire [Questionnaire]	V3.0	22 March 2022
Other [REC provisional responses]	V1.0	22 March 2022
Participant consent form [Patient consent form cover sheet]	V3.0	22 March 2022
Participant information sheet (PIS) [Patient information cover sheet]	V3.0	22 March 2022
Research protocol or project proposal [Protocol]	V2.0	22 March 2022
Summary CV for Chief Investigator (CI) [CV CI]		01 October 2020

Statement of compliance

The Committee is constituted in accordance with the Governance Arrangements for Research Ethics Committees and complies fully with the Standard Operating Procedures for Research Ethics Committees in the UK.

User Feedback

The Health Research Authority is continually striving to provide a high quality service to all applicants and sponsors. You are invited to give your view of the service you have received and the application procedure. If you wish to make your views known please use the feedback form available on the HRA website: http://www.hra.nhs.uk/about-the-hra/governance/quality-assurance/

HRA Learning

We are pleased to welcome researchers and research staff to our HRA Learning Events and online learning opportunities– see details at: https://www.hra.nhs.uk/planning-and-improving-research/learning/

**IRAS project ID: 300134**	**Please quote this number on all correspondence**

With the Committee's best wishes for the success of this project.

Yours sincerely

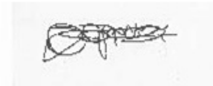

Signed on behalf of;
Mrs Margaret Stephens
Chair
Email: hampshirea.rec@hra.nhs.uk
Enclosures: "After ethical review – guidance for researchers"
